# Insights into conscious cognitive information processing

**DOI:** 10.3389/fnbeh.2024.1443161

**Published:** 2024-07-25

**Authors:** Ekrem Dere

**Affiliations:** ^1^Department of Behavioral and Clinical Neuroscience, Ruhr-University Bochum, Bochum, Germany; ^2^Unité de Formation et de Recherche des Sciences de la Vie (UFR 927), Institut de Biologie Paris-Seine, Sorbonne Université, Paris, France

**Keywords:** incremental learning, discontinuous performance, conscious cognitive information processing, platform theory of conscious cognitive information processing, animal consciousness, behavioral correlates of consciousness

## Abstract

For over a century, the neuro- and pathophysiological, behavioral, and cognitive correlates of consciousness have been an active field of theoretical considerations and empirical research in a wide range of modern disciplines. Conscious cognitive processing of information cannot be observed directly, but might be inferred from step-like discontinuities in learning performance or sudden insight-based improvements in problem solving behavior. It is assumed that a sudden step of knowledge associated with insight requires a creative reorganization of mental representations of task- or problem-relevant information and the restructuration of the task, respectively problem to overcome an cognitive dead-end or impasse. Discontinuities in learning performance or problem solving after an insight event can be used as time-tags to capture the time window in which conscious cognitive information processing must have taken place. According to the platform theory of conscious cognitive information processing, the reorganization and restructuration processes, require the maintenance of task- or problem-relevant information in working memory for the operation of executive functions on these mental representations. Electrophysiological evidence suggests that the reorganization and restructuration processes in working memory, that precede insight-based problem solutions are accompanied by an increase in the power of gamma oscillations in cortical areas including the prefrontal cortex. Empirical evidence and theoretical assumptions argue for an involvement of gap junction channels and connexin hemichannels in cortical gamma-oscillations and working memory processes. Discontinuities in learning or problem solving performance might be used as time-tags to investigate the implication of gap junction channels and hemichannels in conscious cognitive processing.

## Conceptual issues with consciousness

Detailed information on the neurophysiological and molecular mechanisms, as well as regarding the behavioral correlates of consciousness is still scarce (Dere et al., 2021; Zlomuzica and Dere, 2022). This gap of knowledge is even more astonishing in that the keyword “consciousness” entered into the online scientific publication database PubMed returns more than 59.000 hits. Nevertheless, there is no general definition in sight that would be unanimously accepted by all the different disciplines (Dere et al., 2021; Zlomuzica and Dere, 2022). Owed to this conceptual vacuum, the measurement of cognitive, behavioral and neurophysiological correlates of consciousness in animals and humans has been an extremely challenging task. This an untenable situation, if one considers that altered consciousness (e.g., lack of insight into illness, tunnel vision, altered attention, perception and biased processing of disease-relevant stimuli) is a frequent symptom (and sometimes obstacle for successful treatment) among mental, neurological, and psychiatric diseases (Bob et al., 2016; Dere et al., 2021; Zlomuzica et al., 2022; Zlomuzica and Dere, 2022; Martin, 2023; Stefanelli, 2023). However, due to the conceptual difficulty indicated above, alterations in consciousness as a clinical symptom is usually neglected (except in severe cases subsumed under the term disorders of consciousness) where the patient is no longer responsive or oriented in terms of time, location and personal information (Edlow et al., 2021).

## Conscious cognitive information processing

In order to resolve the definition issue and to pave the way to an empiric approach to this phenomenon, one should focus on the adaptive function of consciousness in everyday life and ask, which situations would probably induce a conscious cognitive information processing in the brain. It is reasonable to assume that people engage in conscious cognitive information processing when they are confronted with novel situations in which they cannot apply learned behavior, habits or scripts to master the situation. Such situations can occur, when a problem emerges (for example when environmental reinforcement contingencies suddenly change) and there is no masterplan at hand to cope with such an irregularity.

In contrast, one does not necessarily need conscious cognitive information processing to drive a car, prepare a meal or take a shower. Here, a mental autopilot driven by scripts, habits, reflexive and genetically predetermined behavior, and automatic motor programs would be sufficient to maintain such “unconscious” behavior. However, when something unexpected happens, a new problem is posed, conscious cognitive information processing is required to resolve the situation. Another situation that is likely to require conscious cognitive information processing is mental time travel (Breeden et al., 2016; Dere et al., 2019). People engage in mental time travel to plan for the future, anticipate difficulties and problems and prepare effective coping strategies for such anticipated or imagined problems. Mental time travel into an anticipated or imagined future cannot be successfully implemented without conscious cognitive information processing.

An operational definition of conscious cognitive information processing has been proposed by Dere et al. (2021). The central statement of this definition is that conscious cognitive information processing is initiated, whenever novel situations or problems are encountered that need to be resolved, or when mental time travel is performed to reconstruct past experiences that can be exploited to anticipate, imagine and prepare for future events in order to maximize the probability to experience rewarding situations and to minimize the probability of experiencing aversive situations (Dere et al., 2021).

During conscious cognitive information processing, representations of perceived interoceptive and/or exteroceptive stimuli, as well as related semantic concepts, memories, and experiences are effortfully maintained in working memory to be actively manipulated (e.g., reorganized or restructured) in order to generate a novel creative output. According to this definition conscious cognitive information processing critically depends on the complexity of the situation with which the brain is dealing with and the complexity of the information that is actively manipulated on the working memory workbench (Dere et al., 2021).

## Platform theory of conscious cognitive information processing

**Figure 1:**
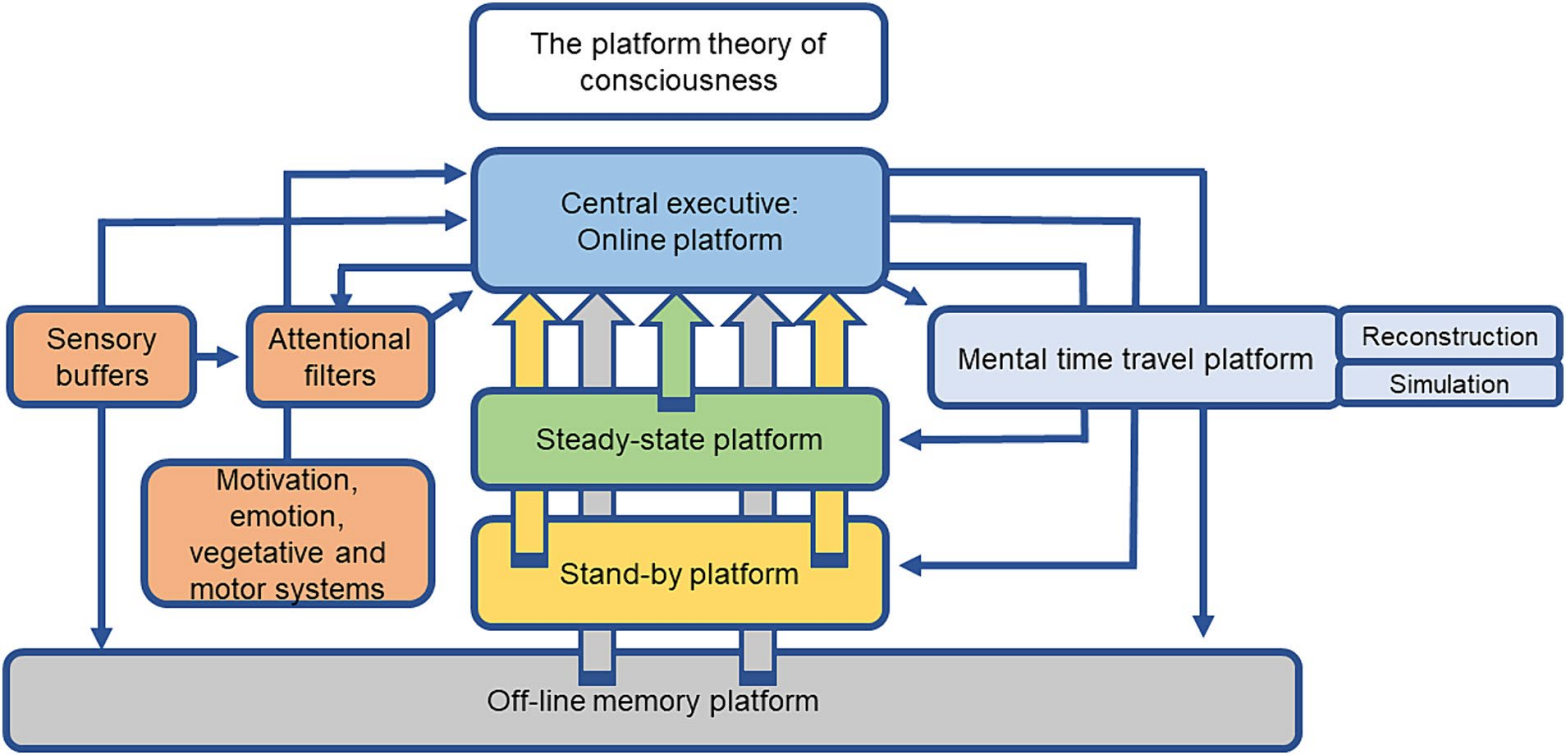
Scheme of the different components of the platform theory of conscious cognitive information processing. The central executive/online platform is a consciousness control center that organizes and monitors conscious mental operations by the orchestration of subordinated operation and storage units called “platforms.” The main function of the central executive/online platform is the generation of a daily schedule or agenda with goals to archive and the allocation of different grades of consciousness to mental representations or contents that are required to accomplish these tasks. The central executive/online platform selects mental representations (internal or external stimuli, stored content from the off-line memory platform) to be maintained, used, manipulated, or to be placed on the steady-state or stand-by platforms. The steady-state, stand-by, and off-line memory platforms do not execute cognitive operations, but rather provide information and content that is important to accomplish tasks or solve problems. The contents of declarative and non-declarative memory including episodic, semantic, and procedural information are stored in the off-line memory platform and are accessible to the central executive/online and mental time travel platforms. The central executive/online platform controls and presets attentional stimuli filters and receives input from sensory systems, as well as from motivation, emotion, vegetative, and motor systems. Intense stimuli can by-pass the attentional filters and directly access the central executive/online platform. The central executive/online platform recruits the mental time travel platform to re-construct past experiences, anticipate future events, or simulate and calculate the outcomes of different actions in imagined scenarios by feeding this platform with information from external and internal perceptions, as well as stored content from the off-line memory platform (Breeden et al., 2016; Dere et al., 2019, 2021; Zlomuzica and Dere, 2022).

The above definition is part of a larger theoretical framework, designed as the platform theory of conscious cognitive information processing, that attempts to explain how conscious cognitive information processing guides and controls flexible, respectively, adaptive behavior in humans and probably animals (Dere et al., 2019, 2021; Zlomuzica and Dere, 2022). The platform theory of conscious cognitive information processing proposes a hierarchical model of perception, memory, cognition and conscious cognitive information processing that is composed of a central executive/online processing platform that serves as a conscious cognitive operation control center that organizes, monitors and orchestrates subordinate operation and storage units called platforms (see Figure 1 for a summary of the platform functions). Conscious cognitive information processing requires the maintenance of mental representations of internal and external stimuli as well as related semantic concepts, memories, and experiences on a working memory platform that is endowed with a multitude of sophisticated executive functions (Breeden et al., 2016; Dere et al., 2019, 2021; Zlomuzica and Dere, 2022). The subordinate platforms serve, for example, as storage media for semantic and episodic knowledge or planned actions, activities, and the working-off of daily agendas.

It has been proposed that the stream of consciousness including conscious perception, conscious processing of information and metacognition relies on the synchronization of neuronal network activity across distant cortical regions (Alkire et al., 2008; Koch et al., 2016; Tian et al., 2020; Valencia and Froese, 2020). These far-reaching synchronization processes are always neuronal mass phenomena with large amplitudes (otherwise the signal could not be filtered out from the background noise), that only vary by the oscillation frequency (Meador et al., 2002; Northoff, 2017). It is difficult to believe that such mass phenomena can represent specific content or cognitive processes. It is more reasonable to assume that they represent more global network synchronization states that enable specific conscious cognitive information processing in local neuronal circuits which in contrast are likely to have an neuronal activity pattern that is sequential, reverberatory, and asynchronous to the global neuronal network synchronization in which these local neuronal circuits are embedded (Singer, 1998; Dere et al., 2021; Zlomuzica and Dere, 2022; Zlomuzica et al., 2023). The activity of local neuronal circuits as singular phenomena cannot be detected with electrophysiological methods such as the EEG and MEG or neuroimaging approaches and require intracerebral and intracellular recordings.

According to the platform theory of conscious cognitive information processing, conscious cognitive information processing and adaptive behavior is supported by local neuronal circuit and neuronal network oscillations shaped by gap junction channels and connexin hemichannels (Dere et al., 2021; Zlomuzica et al., 2022, 2023; Zlomuzica and Dere, 2022). The platform theory of conscious cognitive information processing also proposes a molecular mechanism for conscious cognitive operations in everyday life, including the execution of planned actions, and the accomplishment of behavioral goals and agendas. Specifically, it is proposed that pacemaker cells or neural circuit pulsars embedded into local neuronal circuits and more far-reaching networks (Le Bon-Jego and Yuste, 2007; Wittner and Miles, 2007; Kocsis et al., 2022), connexin hemichannels, and gap junctions jointly generate local neural circuit (and network) oscillations (Draguhn et al., 1998; Coulon and Landisman, 2017; Mäkinen et al., 2018; Traub et al., 2018), which are the basis for the maintenance of mental representations in working memory (Allen et al., 2011), to enable the performance of conscious cognitive operations on these mental representations, and the initiation of behavioral changes, as consequences of these conscious cognitive operations (Dere et al., 2021).

## What is the “added value” of the platform theory of conscious cognitive information processing compared to other empirical and theoretical approaches to consciousness?

There is a great deal of research that attempts to correlate very general “states” of consciousness with corresponding “levels” of consciousness. These states include coma, vegetative state (unresponsive wakefulness syndrome), minimally conscious state, sleep and the awake state (Laureys et al., 2010). However, since coma and vegetative state are usually the consequence of a severe disease, injury or poisoning, they are essentially manifestations of brain dysfunction or breakdown, rather than states of consciousness. The inability to maintain consciousness after brain damage or the absence of consciousness in a diseased brain should not be considered as a state of consciousness or a condition that should be investigated to understand the nature of consciousness in an intact and operational brain. If one follows such a rationale until its end, death could be also regarded as a state of consciousness that is associated with an “absolute zero” level of consciousness.

Attempts have also been made to determine the neural basis of consciousness through imaging and electrophysiological measurements during the regaining or recovery of consciousness, for example after anesthesia or upon awakening (Edlow et al., 2021). The regaining of consciousness has been related to the “re-boot” of resting state networks including the default mode network, fronto-parietal network, and the salience network (Li et al., 2023). At most, these measurements can be used to distinguish between a waking receptive state (and the brain networks required) and a non-receptive state, but they do not say much about brain processes that are associated with conscious cognitive information processing (see also Chenot et al., 2024). To use a metaphor, it’s like measuring the lowering of a rolled up ceiling recessed projector screen to project content onto it. It does not give much information about the projector or the content that is being projected.

Yet, another line of research probes the awake brain to tease apart conscious and unconscious processing. This seems to be a more convincing and promising approach which is also followed by the platform theory of conscious cognitive information processing. According to this line of research, conditions, respectively, experimental situations have to be created, in which conscious cognitive information processing is likely to be initiated and therefore can be measured. In other words, the “brain” can be actively put in a situation which “inevitably” initiates conscious cognitive information processing and can be “observed” or “measured” during conscious cognitive information processing. In the context of the platform theory of conscious cognitive information processing, consciousness is treated as an observable variable and it is proposed that conscious cognitive information processing is initiated in situations where the owner of the brain is confronted with a novel problem for which there is no prefabricated solution available. Consequently, it is assumed that, at the moment of an insight-based solution, the brain was engaged with conscious cognitive information processing, which is different from unconscious information processing. In other words, the changes in the electro- and neurochemical activity of different areas of the brain that accompany this special moment of insight will certainly tell us much more about the neurophysiological mechanisms of conscious cognitive information processing than the comparison of functional network activity during pathological brain states including the unresponsive wakefulness syndrome and minimally conscious state (Panda et al., 2022).

Furthermore, there are reductionist approaches that attempt to identify electrophysiological correlates for the conscious subjective perception of visual stimuli (in contrast to basic activity that is evoked by mere sensory stimulation) in order to gain insight into neurophysiological mechanisms and principles which might also help to understand more complex phenomena, including conscious cognitive information processing. Animal research in this area has suggested that visual awareness is reflected by power modulation of high-frequency local field potentials (in the gamma oscillation range) in the lateral prefrontal cortex, temporal and parietal cortex, where spiking activity is found to be perceptually modulated (Panagiotaropoulos et al., 2012; Tseng et al., 2016). The gamma oscillations in this brain network might be related to the maintenance of “conscious visual perceptions” in working memory (or online platform) for further conscious cognitive information processing in order to generate a novel creative output. In this regard these visual consciousness approaches emphasize the importance of working memory for the generation and maintenance of conscious cognitive information processing, in a similar way to the platform theory of conscious cognitive information processing (Dere et al., 2021).

One of the most influential theories of consciousness is Bernhard J. Baars’ Global Workspace theory (updated in Baars et al., 2021). It attempts to explain how a serial, integrated and very limited stream of consciousness emerges from a system of specialized “receiving processes” that are “unconsciously” working in parallel. The most peripheral “receiving processes” transmit information from primarily sensory pathways to downstream “receiving processes” that process and interpret these sensory information. The content entering the global workspace is then projected to secondary cortical sensory association areas. It is proposed that only the most significant information is allowed to enter the global workspace, which in turn can be regarded as a fleeting memory area with a limited storage capacity (in the range of seconds). It is assumed that only information that is passively stored (for a few seconds only) within the global workspace is conscious. “Consciousness” is regarded as a passive response (the information in the global workspace is not subject to cognitive operations) to a significant and intense sensorial stimulation. Formulated pointedly, the global workspace theory merely describes a sensory gripping reflex without specifying the neuronal or molecular mechanisms that underlie this function (Zlomuzica and Dere, 2022). The global workspace is therefore not well suited to maintain a larger amount of information which might be required to solve complex problems. It is therefore not clear how this model can explain conscious cognitive information processing, complex problem solving or mental time travel into the past and future in any way better that the platform theory of conscious cognitive information processing (see also Zlomuzica and Dere, 2022).

Another popular theory of consciousness is the integrated information theory, since it provides a mathematical model to actually measure the level of consciousness in any neuronal network or other system that is potentially capable to generate consciousness (Tononi et al., 2016). In the framework of this theory it is assumed that consciousness is a subjective, immediate, direct, and unified process. It further attempts to define the basic properties of a physical system capable of generating consciousness. According to this theory, consciousness necessities an interconnected set of elements with reentrant feed-back loops, in which the single elements have a mutual physical cause-effect powers on each other leading to the integration of information. The integrated information theory equates integrated information with consciousness, suggests that the degree of consciousness (in both quantity and quality) is measurable by determining the amount of intrinsic cause-effect power via phi metrics and extends its claims beyond human consciousness to animal and artificial consciousness. The integrated information theory attempts to define the necessities and operation mode of physical substrates of consciousness in the process of generation of integrated information and system-immanent intrinsic modulation. Testing the compatibility of this theory with the platform theory of conscious cognitive information processing would require measuring the postulated cause-effect system-immanent intrinsic-modulation during the execution of a conscious cognitive information processing or insight event. It is currently difficult to imagine what such a proof-of-principle experiment should look like. In a first step, one could try to detect corresponding cause-effect activity patterns or self-modulating systems in an organic model system such as brain slice preparations from animals or brain organoids. As promising and intuitively plausible as this theory may seem at first glance, the essential assumptions of the theory are essentially claims that have no empirical basis and are hardly amenable to experimental verification in a healthy, intact and awake brain.

### Conscious cognitive information processing in animals

Despite the importance of conscious cognitive processing for the mental life and adaptive behavior of humans (and with limitations in animals), and in view of the devastating consequences of impaired conscious cognitive processing in patients with mental disorders and neuropsychiatric diseases, there is no valid animal model of conscious cognitive information processing available, that could be exploited for psychopharmacological, comparative and translational research (Zlomuzica et al., 2022, 2023; Dere and Zlomuzica, 2023). Animal models of conscious cognitive information processing have to deal with important issues of construct, face and predictive validity (Dere et al., 2021; Zlomuzica and Dere, 2022). In a recent review, the few available paradigms to measure animal consciousness have been reviewed and the conclusion was reached, that all available tests have major shortcomings and are not well suited to serve as routine tests for neurobiological and pre-clinical research (Zlomuzica and Dere, 2022). Instead the combination of a number of sophisticated cognitive tests in a behavioral test battery and the calculation of individual composite performance scores might be a better approximation to the measurement of conscious cognitive information processing. However, the proposed test battery is very time consuming, requires considerable equipment, training, and expertise with behavioral testing. These factors will probably hinder the application this test battery on a larger scale. There might be an “cheaper” and more direct path to conscious cognitive information processing in animals and humans.

### Insight

Classic and contemporary definitions of the insight phenomenon by cognitive psychologists conceptualize insight as a sudden comprehension, realization, or creative problem solution that is based on a reorganization of mental representations of relevant information that is opposed to incremental trial-and-error learning (Hebb, 1949; Thorpe, 1956; Kounios and Beeman, 2014; but see Bowden et al., 2005 for a different view). A sudden insight into the nature of a problem that leads to an instantaneous step of knowledge is likely to be accompanied by a sudden and strong emotional arousal (Shen et al., 2017) resulting in the formation of an episodic memory (Dere et al., 2006, 2010; Kinugawa et al., 2013; Pause et al., 2013), which probably from this point on is the mnemonic basis of the persisting improvement in task performance.

In Wolfgang Köhler’s insight learning theory (Köhler, 1917, 1925), insight is defined as an instantaneous type of understanding of relations and reinforcement contingencies that can emerge without prior trial-and-error learning and that leads to a solution to a current problem. Learning success based on insight has been proposed to be conceptually incompatible with learning based on operant conditioning or reinforcement learning which is associated with a gradual buildup of a reward value or reinforcement signal for the correct response or sequence of responses accumulated through experiences (Thorndike, 1911; Schultz et al., 1997).

### Insight and conscious cognitive information processing

Event-related potential studies suggest that complex perceptions and cognitive processes can occur in the range of milliseconds. It has been proposed that a sudden step of knowledge might also be the result of a latent process that that runs unconsciously in the background and suddenly reaches consciousness while escaping metacognitive monitoring up to this point of time (see Tolman, 1948; Qiu et al., 2008).

There is an ongoing debate on whether insight is the result of conscious information processing (in the form of progress monitoring) after it has been realized that conventional solution attempts will not create the goal configuration or attenuate the problem space, e.g., in the 9-dot problem (MacGregor et al., 2001) or an unconscious process in which self-imposed constraints on a problem or misleading presuppositions are discarded, and chunked items in the problem are decomposed and redistributed (Knoblich et al., 2001).

There are also many anecdotical stories of sudden “inspirations” of how great researchers and scholars suddenly had an idea which immediately solved a problem that they had been trying to solve for a long time (think of the apple falling on Newton’s head or Archimedes idea to calculate material density through water displacement). The truth content of these romanticized stories of great scientists and inventors might be questionable. Nevertheless, many people report similar experiences of “inspiration” defined as a non-religious or mystical thought or idea that suddenly arises, is recognized as a solution to a problem, and seems to be detached from the actual context and current stream of thoughts or thinking.

It is not plausible to assume that an inspiration comes out of “nothing,” i.e., without preparatory cognitions. It seems for example more plausible to assume that conscious cognitive processing of information took place in advance, but the end product of this reasoning has not been recognized as the solution (and therefore has been “put aside”). It is also possible that in the course of reasoning, initially a small fragment of the solution was missing, but has been added at a later point in time. Phenomenologically, these processes might be felt as a flash of inspiration propelled by an metacognitive illusion (Dere et al., 2021). Therefore, it seems to be more reasonable to assume that an “inspiration” is probably the intrusion of an insight-based solution (or promising part of the solution after a period of “incubation”), that has not been acknowledged as such by the time it was generated. For the remainder of this article insight is defined as the end product of an ongoing conscious cognitive information process, which should not be equated with the term “inspiration.”

### Examples for insight-based problem solving in humans, non-human primates, and laboratory rats and mice

Robert W. Weisberg proposed in his integrated theory of insight in problem solving, that insight depends on conscious cognitive operations that aim to restructure problem-relevant information, in a way that the new information structure (comparable to individual puzzle pieces that have been put together), enables a direct solution to the problem (Weisberg, 2015). Insight can be regarded as the endpoint or manifestation of a hidden problem solving mechanism and has been studied, for example, with ill-structured innovation tasks, including the hook bending paradigm or the tower of Hanoi. These tasks have in common that they require a multi-step solution, whereby the solution path is unknown and only information about the goal or target configuration is provided. Children who were at least 7 years old usually arrived suddenly at the solution to the hook bending problem, suggesting an insight-like problem solving mechanism (Defeyter and German, 2003; Cutting et al., 2014). The hook bending problem has also been posed to non-human primates and large brained birds, which both showed task performance (e.g., very few failures after the task has been solved for the first time) that was interpreted in the sense of tool innovation that was made possible through sudden insight into the problem (Weir, 2002; Bird and Emery, 2009; Laumer et al., 2018).

The Gestalt- and comparative psychologist Wolfgang Köhler investigated the phenomenon of insight in nonhuman primates (Köhler, 1917, 1925). The food-deprived chimpanzee Sultan had to realize that two small sticks in the cage could be inserted into each other to be able to pull a banana within reach that had been placed outside the cage. After several unsuccessful attempts to reach the banana with one of the two sticks, Sultan managed to put the two small sticks together by accident. Sultan immediately took the stick and pulled the banana towards him. However, this behavior cannot be clearly interpreted as insight because the solution was found more or less by chance. Nevertheless, Sultan understood that he needed a longer stick and when he saw it, he considered the problem solved and went straight to implementing it (Köhler, 1917). However, it must be noted that the experiment was probably not designed in an ecologically valid way. In the wild, respectively, nature there are usually no sticks that can be stuck together, just like there are no magic wands. Therefore, Sultan would possibly not have been able to find the solution right away through prior conscious cognitive processing of all the information available. However, it is quite possible that Sultan, as a result of conscious cognitive processing of information, came to the conclusion that only a stick twice as long would make it possible to pull the banana.

As previously mentioned, insight can emerge from reorganizing or restructuring information (MacGregor et al., 2001; Weisberg, 2015). However, this cognitive process can be impaired if a so-called functional fixation is present (McCaffrey, 2012). This means, for example, that a tool is very strongly associated with a certain type of use or activity, so that a potential unconventional use of it to solve a problem is masked. Consequently, it has been shown that great apes have difficulties to find a solution to a new problem, when the available tools have been strongly associated with a different type of use or the solution of another problem (Ebel et al., 2020). Again task design seems to be highly critical for the usefulness or sensitivity of a task or paradigm to detect problem solving based on insight.

The first study into the question of whether lower mammals such as rats are able to solve problems through insight was published by Helson (1927). In the first phase of an elegant discrimination learning experiment, Helson trained one pair of rats (first pair) to prefer a food-rewarded light (60 W illumination) over a non-rewarded dark compartment (15 W illumination) and another pair of rats (second pair) to show the opposite preference. In the second phase of the experiment the illumination intensity of the two compartments was changed to 150 W and 60 W for the first pair of rats and to 15 W and 1 cd for the second pair of rats, while the reward contingency or task rule was not changed. The first pair would receive a reward for choosing the lighter compartment, while the second pair would receive a reward for the darker compartment. After the change of the intensity values of the stimuli presented the rats continued to prefer the light, respectively, dark compartment, suggesting that their decision was guided by structure–function relationships rather than simple stimulus–reward associations. The latter would require the rats to stay with the initially rewarded stimulus rather than to switch immediately to a novel stimulus that has not been paired with a reward. Helson concluded that the adaptive behavior of the rats was based on insight into the structure or general rule of the task (Helson, 1927).

### Behavioral correlates of insight as a manifestation of conscious cognitive information processing

Insight is a singular phenomenon that cannot be reliably reproduced over multiple teaching sessions and it can only be examined at the level of an individual and not in groups (but see Zlomuzica and Dere, 2022). Conscious cognitive processing of information cannot be observed directly but can perhaps be inferred from discontinuities in learning or problem solving behavior. As indicated above conscious cognitive information processing is initiated whenever a novel problem is posed that cannot be solved through learned, instinctive, or reflective behavior (Dere et al., 2021; Zlomuzica and Dere, 2022) and it is realized that there is no ready-made solution available.

A new problem might be initially addressed through trial-and-error learning (Thorndike, 1911). This type of problem solving strategy depends on randomly generated actions or sequences of actions. One of which happens to be correct and brings about the desired solution to the problem. Trial-and-error learning is generally characterized by gradual, incremental or continuous learning (Thorndike, 1911). Even after a correct action or sequence of actions has been executed and the problem has been temporarily solved, it is possible that the correct response to the problem is not remembered shortly after. Just imagine that you incidentally managed to solve a “computer or software problem,” there is no guarantee that you will remember the sequence of actions that you have performed the next time when you are confronted with the same or a similar problem. The trial-and-error learning process may thus be more tedious than a quicker solution based on insight. The latter by definition is characterized by discontinuous or step-like learning and a sudden “step of knowledge” which stamps in permanently the correct response to the problem. The main point of this review is the hypothesis that learning and associated abrupt changes in performance through insight are likely to be the consequence of previous conscious cognitive information processing and that sudden and persistent changes in learning performance (discontinuities in learning performance) can be used as time tags indicating the time windows in which conscious cognitive information processing must have taken place. Equally, it can be assumed that an individual who exhibits merely continuous learning without abrupt increases in performance has not initiated conscious cognitive information processing to solve the problem.

**Figure 2:**
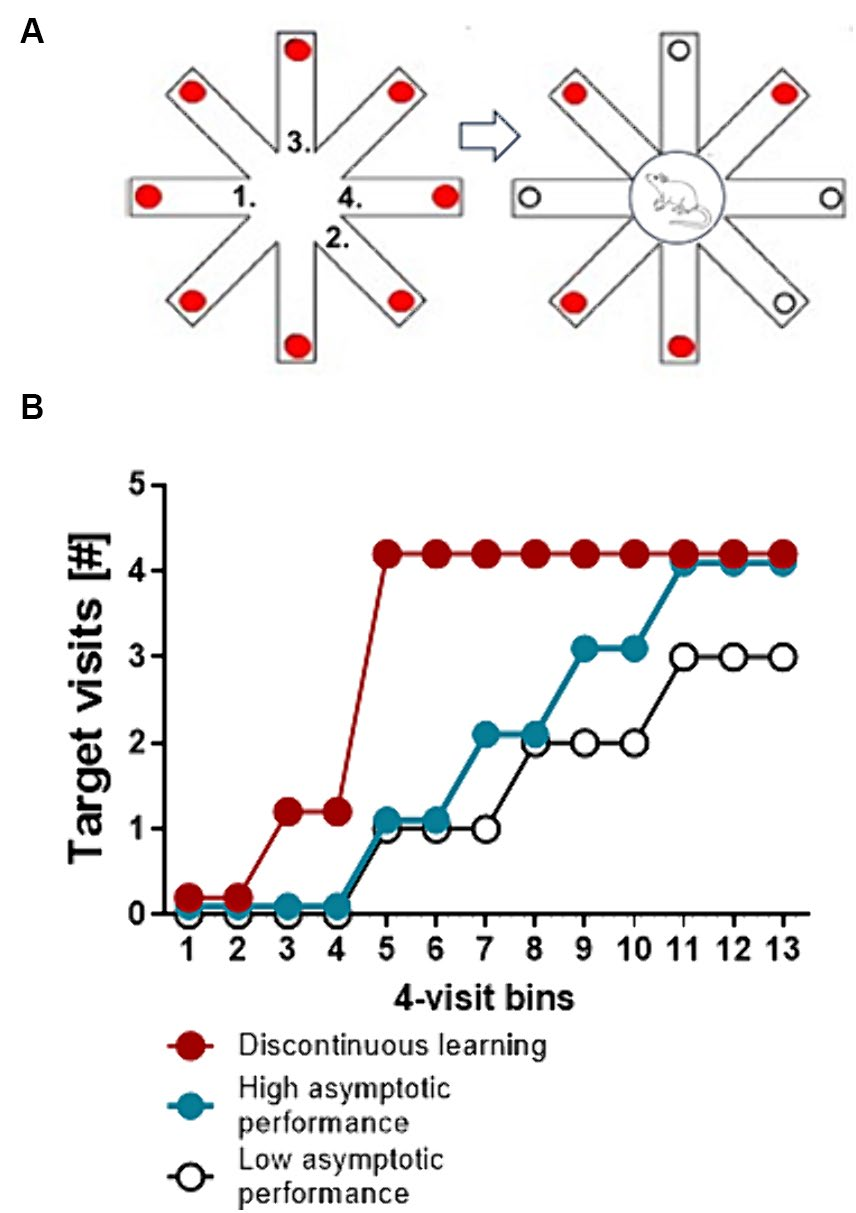
Discontinuous and continuous learning performance. **(A)** Schematic drawing of a simple spatial working memory test to identify continuous and discontinuous learning performance. Depicted is a food-rewarded radial eight-arm maze. Red circles represent food rewards. The test starts with a free choice trial, in which a food deprived animal is allowed to make 4 choices. An example of a sequence of choices is indicated by the numbers. After the 4^th^ choice the animal is trapped at the central platform for a retention interval (e.g., 10 s). Thereafter all doors are unblocked and the animal is allowed to make 4 more choices. The number of correct choices in 4-choice bins (target choices of arms which are still baited) is recorded. **(B)** Line and scatter plots represent idealized learning curves (discontinuous learning, fast and slow incremental learning) of individual mice. Discontinuous learning is characterized by a sudden and stable improvement in the number of correct target choices within 4-choice or corner visit bins. Continuous learning is characterized by gradual incremental improvements in performance. The speed of learning equals the slope of the curve while a learning impairment is indicated by low asymptotic performance.

Translated into an experimental learning paradigm this sudden insight would be reflected by an equally sudden and stable improvement in performance (Gallistel et al., 2004). The analysis of the performance dynamics of individual participants or experimental animals during the acquisition of a task can help to identify the time point of this discontinuity in performance and thus the time point when conscious cognitive processing has been initiated. On the other hand the analysis of performance dynamics can also differentiate between slow and fast incremental continuous learning, that is the distinction between superior and inferior learners that do not engage in conscious cognitive processing and thus do not show discontinuities in learning performance (Figure 2).

In a recent experiment by Rosenberg and colleagues, mice that had to solve a complex labyrinth navigation problem showed “sudden insight” that is a sharp discontinuity during learning, when the mice figured out to take a long but direct path with more than 6 correct binary choices to the goal. This sudden improvement in task performance persisted for the remainder of the experiment (Rosenberg et al., 2021). Rosenberg et al. (2021) developed a “sudden insight statistics” procedure with which the slope of an individual learning curve can be analyzed in a way that enables the distinction between individuals that show a discontinuous learning curve from those that show continuous or incremental learning. This work demonstrates that the insight phenomenon can be quantified at the level of individual behavior and statistically analyzed (see also Reddy, 2022).

In conclusion, insight and associated discontinuities in learning or problem solving behavior can be regarded as the successful endpoint, respectively, outcome of conscious cognitive information processing, that might be used to time tag conscious cognitive processing and to investigate the underlying neurophysiological processes and substrates. This is especially relevant for experiments where learning behavior and electrophysiological readouts are measured simultaneously and continuously. It should be noted that has also been suggested that discontinuities in learning behavior do not necessarily reflect insight-based problem solving, but might be the consequence of other cognitive processes (Van Steenburgh et al., 2012). However, this uncertainty might be eliminated by demonstrating the specificity of a putative neuro- or electrophysiological readout or correlate of discontinuous learning. For example, in the context of electrophysiological measurements, one could identify neuronal oscillations of certain frequency bands and power that correlate temporally with jumps in performance and that are not found in a comparable form or intensity during continuous learning. Future research will determine whether the neurobiological basis of conscious cognitive processing can be tackled by the simultaneous measurement of insight-like discontinuities in learning performance and *in-vivo* electrophysiological recordings and/or optogenetic manipulations with high temporal resolution.

### The neurophysiological basis of discontinuous learning based on sudden insight

In a visual face and place recognition experiment with non-human primates, it has been found that discontinuous learning performance or sudden steps of knowledge coincides with a transient peak in neuronal network synchronization between the reward-sensitive areas of the prefrontal cortex and inferotemporal cortex known for the processing of images (Csorba et al., 2022). However, the authors also reported that the amplitude of cross-region synchronization increased gradually across task performance, so that the maximal synchronization was build up slowly and not suddenly. These findings suggest that discontinuous or step-like learning performance based on insight might require the synchronization of neuronal activity in the brain areas that are involved in the processing of task-relevant information. Given that the synchronization of cross-regional neuronal activity was build up gradually, it is tempting to speculate whether conscious cognitive information processing that eventually leads to an sudden improvement in task performance is reflected by a gradual and cumulative increase in the synchronization of neuronal activity in the brain regions involved in the mental representation, maintenance and processing of task-relevant information.

Further evidence for the involvement of the prefrontal cortex in discontinuous learning performance that might be based on an insight-like decision process comes from a rule-shifting task with rats (Durstewitz et al., 2010). In this experiment, rats were first trained to acquire a cue-based response strategy to obtain rewards in an operant chamber. Thereafter, the rats experienced a change in the reinforcement contingencies that required them to abandon the old strategy and gradually acquire a different egocentric response strategy by gathering evidence through trial-and-error. The authors suggested that novel rule learning can be regarded as an evidence-based decision process, that might be accompanied by moments of sudden insight at the instant, when there is enough information gathered that undoubtedly indicates that the reinforcement contingencies in the learning situation have changed permanently (a similar interpretation can be found in Bowden et al., 2005). It was found that transitions in behavioral performance of rats during rule learning in a set-shifting task were temporally correlated with abrupt transitions in the firing activity of “rule-selective” neural ensembles in the prefrontal cortex (Durstewitz et al., 2010). This study suggests that behavioral and neural dynamics can be correlated in gradual or incremental learning situations, where an inefficient response gradually decreases, while an efficient response gradually increases. However, it remains to be determined whether the detected correlation between behavioral and neural dynamics in this experiment indeed reflects an insight-like cognitive process, or whether it is the manifestation of the execution of different responses, respectively the active inhibition of the inefficient response. It should be also considered that, one important criterion for the appreciation that a change in behavior occurred after insight, is that the performance leap is permanent, illustrated by a step-like learning curve (without a transition phase), which was probably not the case in the study by Durstewitz et al. (2010).

A combined scalp electroencephalogram and functional magnetic resonance imaging study by Jung-Beeman and colleagues searched for the neural correlates of insight-like verbal problem solving. In this experiment, participants had to state whether the correct solutions were reached by insight or not. Insight-based solutions were found to be preceded (0.3 s prior to insight solutions) by a sudden burst of neural oscillations in the gamma-frequency range in the anterior superior temporal gyrus of the right hemisphere. Insight solutions also associated with increased neuronal activation in the anterior superior temporal gyrus as compared to non-insight solutions (Jung-Beeman et al., 2004).

Further support for the implication of gamma-frequency neuronal oscillations in insight-like problem solving was provided by an electroencephalogram study by Rosen and Reiner (2017). Here, participants were asked to solve a spatial puzzle (which can be solved either incrementally or by insight), and had to indicate whether they found the solution by sudden insight or in another way. Participants who have reported solutions based on insight showed an increase in gamma and beta frequency activity in frontal areas and with respect to alpha frequency activity in right temporal areas as compared to participants who reported an solution based on incremental learning (Rosen and Reiner, 2017). Interestingly, the incremental group exhibited a decrease in gamma and beta activity during the task performance as compared to baseline recordings, suggesting that different solution strategies are mediated by different neuronal network operations rather than on the basis of a gradual difference. The latter alternative would mean that both an insight-based solution and a solution based on incremental learning would require gamma activity, but an insightful solution would be of example only possible if a certain intensity or power threshold is exceeded. In conclusion, these findings suggest that the assumed reorganization of information and the restructuration of the spatial puzzle problem prior to the insight-based solution was associated with gamma frequency neural oscillations in the right frontal cortex.

Another electroencephalographic study searched for neural activity that might be specific for different phases of insight-based problem solving, including a mental impasse (when it is realized that routine solutions are useless), the reorganization of the relevant information to have a different access to the problem, and, finally the sudden insight into the problem and its solution based on subjective ratings (Sandkühler and Bhattacharya, 2008). According to the authors the states of mental impasse or sudden insight were correlated with the power of gamma-frequency activity at parietal-occipital regions, while the reorganization of task-relevant information seemed to be associated with a decreased power in the alpha-frequency range in the right prefrontal area (Sandkühler and Bhattacharya, 2008).

In the studies reported above, the categorization of insight vs. non-insight solutions has been made by subjective or introspective reports requiring metacognition (hereinafter referred to as insight-like problem solving), rather than on the basis of more objective criterions, such as discontinuous learning performance as suggested above. Therefore, it remains to be determined whether the findings reported above can be replicated when a behavioral criterion for insight-like problem solving is used as a specifier for problem solving based on insight.

The studies discussed so far suggest an involvement of cortical brain regions in insight-like problem solving (prefrontal cortex, anterior superior temporal gyrus, parieto-occipital regions showing neural activity in the gamma-frequency range). However, there is also evidence for a subcortical contribution to insight-like problem solving. In an functional magnetic resonance experiment, participants signaled correct solutions in a remote associates task (inferring the commonality in word triplets) with a button press, which has been used as a time-tag for sudden insight-like solutions (Tik et al., 2018). Insight-like solutions were not only accompanied by neuronal activation in the left anterior middle temporal gyrus, but also by bilateral activations in the thalamus, hippocampus, ventral tegmental area, nucleus accumbens, and caudate nucleus. The authors conclude that the subjective feeling of relief and the emotional arousal induced after the sudden emergence of the solution is accompanied by a reward signal in the mesolimbic dopamine system (Tik et al., 2018). It would be interesting to know, whether insight-based solutions of more complex problem configurations (e.g., after conscious cognitive information processing) would likewise be accompanied by a subcortical dopaminergic reward signal. Such a reward signal is normally associated with strong emotional arousal, that is known to be required to trigger episodic memory formation for the preservation of the correct solution (Dere et al., 2006, 2008, 2010; Pause et al., 2013). This episodic memory for the problem solving event is very likely the mnemonic basis for the persistent improvement in task performance that follows after problem solving based on insight.

### The role of gap junction channels and hemichannels for discontinuous learning based on insight

Electrophysiological evidence suggests that the reorganization and restructuration processes in working memory, that precede insight-based problem solutions are accompanied by an increase in the power of gamma oscillations in cortical areas including the prefrontal cortex. In the following, I will review evidence for the involvement of gap junction channels and connexin hemichannels in cortical gamma-oscillations (Cunningham et al., 2004; Traub and Whittington, 2010, 2022; Traub et al., 2020) and working memory processes.

Gap junction channels composed of connexin proteins allow an intercellular coupling between neighboring cells. The connexins Cx36 and Cx45 are expressed in neurons, while Cx43 and Cx30 are expressed in astrocytes (see Dere, 2013 for an overview). Intercellular electrotonic and metabolic coupling allows the bidirectional diffusion of ions, cations, metabolites, second messengers, cyclic nucleotides, oligonucleotides, and small molecules with a molecular mass up to 1–2 kDa (Dere, 2013). Coupling and uncoupling of adjacent cells via gap junctions is activity-dependent, shows plasticity similar to the modulation of synaptic strength, and is not restricted to cells of the same type (Yang et al., 1990; Landisman et al., 2002; Alev et al., 2008). In this way, neuronal depolarization can theoretically directly propagate across neurons and generate action potentials. Intercellular communication and signal transmission via gap junctions operates almost without a temporal delay and is therefore much faster as compared to synaptic neurotransmission that operates at least in the order of milliseconds (Dere and Zlomuzica, 2012; Dere, 2013).

Connexin hemichannels and gap junctions have been shown to shape neuronal network oscillations throughout the brain (Coulon and Landisman, 2017). These network oscillations appear to be critically involved in working memory, cognition, and behavior (Allen et al., 2011; Maciunas et al., 2016; Walrave et al., 2016; Tao et al., 2021; Linsambarth et al., 2022). There is evidence that theta and gamma oscillations in hippocampal neuronal networks are mediated by gap junction channels composed of Cx36, Cx45, or both (Hormuzdi et al., 2001; Schmitz et al., 2001; Meier et al., 2002; Buhl et al., 2003; Zlomuzica et al., 2010). Electrophysiological studies with Cx36 deficient mice revealed slower hippocampal theta oscillations (Allen et al., 2011), reduced overall power, and synchrony of hippocampal gamma oscillations *in vitro* (Hormuzdi et al., 2001), and *in vivo* (Buhl et al., 2003). Furthermore, the administration of gap junction blockers induced a complete suppression of hippocampal gamma oscillations in Cx36-deficient mice (Buhl et al., 2003). Furthermore, Cx45-deficent mice showed changes in kainite-induced gamma oscillation in the CA1 and CA3 region together with impaired performance in an object recognition task (Zlomuzica et al., 2010).

The behavioral phenotyping mCx36 deficient mice in our lab revealed changes in motor coordination and balancing performance, increased locomotion and running speed in a novel environment, changes in Y-maze exploration, increased anxiety-related behavior; changes in novel object exploration and impaired delay-dependent object and object-place recognition (Frisch et al., 2005; Zlomuzica et al., 2012). The behavioral phenotypes of Cx36 and Cx45 deficient mice suggest altered learning and memory performance (including behavioral and emotional habituation to normal environments in Cx36-deficient mice) that might be related to changes in theta and gamma oscillations and conscious cognitive information processing.

Given that intercellular communication via gap junctions shows activity-dependent plasticity and might contribute to contribute to the formation of functional cell assemblies (Traub et al., 2020), it would be extremely interesting to find a way that would allow the monitoring of the cellular coupling/uncoupling status of cells in the prefrontal cortex, before and after behavioral discontinuities during learning performance and problem solving. It is tempting to speculate that the reorganization of information and restructuration of the problem faced, might be associated with the sudden coupling or uncoupling of neuronal circuits and/or networks via gap junctions that ultimately leads out of an impasse or cognitive dead end and towards a sudden insight.

The platform theory of conscious cognitive information processing holds that the conscious experience of a mental representation requires working memory. The neuronal mechanism underlying working memory is thought to be a sustained reverberatory neuronal activity in the neural circuit that contains the mental representation. This maintained mental representation can then be used and manipulated by the central executive/online platform (Dere et al., 2021). The platform theory of conscious cognitive information processing further proposes that the reverberation in the neuronal circuits could be maintained by the help of gap junction channels between neurons, astrocytes, as well as undocked connexin hemichannels (Rash et al., 2001; Nagy et al., 2004; Dere et al., 2021). Successive neurons in the neural circuit can be either directly coupled via gap junctions or might be both coupled to the same astrocyte. Continuous reverberation and sustained excitability in neuronal circuits depends on the fast redistribution of ions and metabolites between the cytoplasm and extracellular space. These adjustments might be ensured by connexin hemichannels (Dere et al., 2021). Connexin hemichannels are undocked connexin channels in the plasma membrane that are involved in paracrine communication. They provide a conduit between the intracellular and extracellular space, allowing the passage and exchange of ions and metabolites between the cytosol and extracellular milieu (Dere and Zlomuzica, 2012; Dere, 2013). Therefore, gap junctions and connexin hemichannels have biophysical characteristics that are well suited to support working memory through fast and continuous propagation of neuronal depolarization between neurons in the neuronal circuit (probably mediated via gap junction channels with astrocytes) and via the preservation of excitability of the individual neurons for prolonged periods through the regulation of intra and extracellular ion homeostasis (ensured by the unopposed hemichannels; Dere et al., 2021).

## Conclusion

The empirical evidence and theories presented so far can be summarized into 10 basic assumptions which might be helpful for future research into the neurophysiological mechanisms of conscious cognitive information processing.

1. Insight is the endpoint of conscious cognitive information processing, and, at the behavioral level, leads to a sudden step-like discontinuity in learning or problem solving performance.
2. Insight is based on a creative reorganization of mental representations of task-relevant information and the restructuration of the problem to overcome an impasse.
3. The reorganization and restructuration process requires the maintenance of task-relevant information in working memory and the operation of executive functions on these mental representations (ensured the central executive on the online platform).
4. The reorganization and restructuration processes, which precede sudden insight are correlated with an increase in the power of gamma oscillations in the prefrontal cortex, anterior superior temporal gyrus, parieto-occipital regions.
5. Gap junctions and connexin hemichannels have been implicated in cortical network oscillations and might be involved in the oscillation changes that are associated with conscious cognitive processing and insight.
6. On a subjective level, insight is perceived as a sudden comprehension, realization, or creative problem solution.
7. The subjective feeling of relief and the emotional arousal experienced by the sudden emergence of the solution might be accompanied by a reward signal in the mesolimbic dopamine system.
8. Insight is associated with strong emotional arousal that triggers episodic memory formation creating a memory for the insight event that includes context and content (solution path) information.
9. The generated insight memory is the basis of the persistent improvement in task or problem performance that is recollected whenever the same or similar problems are encountered.
10. Discontinuities in learning or problem solving performance might be used as time-tags to investigate the implication of gap junction channels and hemichannels in conscious cognitive processing.

## Data availability statement

The original contributions presented in the study are included in the article/supplementary material, further inquiries can be directed to the corresponding author.

## Author contributions

ED: Conceptualization, Methodology, Writing – original draft, Writing – review & editing.

## References

[ref1] Alev C. Urschel S. Sonntag S. Zoidl G. Fort A. G. Höher T.. (2008). The neuronal connexin-36 interacts with and is phosphorylated by CaMKII in a way similar to CaMKII interaction with glutamate receptors. Proc. Natl. Acad. Sci. USA 105, 20964–20969. doi: 10.1073/pnas.0805408105, PMID: 19095792 PMC2605416

[ref2] Alkire M. T. Hudetz A. G. Tononi G. (2008). Consciousness and anesthesia. Science 322, 876–880. doi: 10.1126/science.1149213, PMID: 18988836 PMC2743249

[ref3] Allen K. Fuchs E. C. Jaschonek H. Bannerman D. M. Monyer H. (2011). Gap junctions between interneurons are required for normal spatial coding in the hippocampus and short-term spatial memory. J. Neurosci. 31, 6542–6552. doi: 10.1523/JNEUROSCI.6512-10.2011, PMID: 21525295 PMC3160467

[ref4] Baars B. J. Geld N. Kozma R. (2021). Global workspace theory (GWT) and prefrontal cortex: recent developments. Front. Psychol. 12:749868. doi: 10.3389/fpsyg.2021.749868 34899489 PMC8660103

[ref5] Bird C. D. Emery N. J. (2009). Insightful problem solving and creative tool modification by captive nontool-using rooks. Proc. Natl. Acad. Sci. USA 106, 10370–10375. doi: 10.1073/pnas.0901008106, PMID: 19478068 PMC2700937

[ref6] Bob P. Pec O. Mishara A. L. Touskova T. Lysaker P. H. (2016). Conscious brain, metacognition and schizophrenia. Int. J. Psychophysiol. 105, 1–8. doi: 10.1016/j.ijpsycho.2016.05.003, PMID: 27178724

[ref7] Bowden E. M. Jung-Beeman M. Fleck J. Kounios J. (2005). New approaches to demystifying insight. Trends Cogn. Sci. 9, 322–328. doi: 10.1016/j.tics.2005.05.012, PMID: 15953756

[ref8] Breeden P. Dere D. Zlomuzica A. Dere E. (2016). The mental time travel continuum: on the architecture, capacity, versatility and extension of the mental bridge into the past and future. Rev. Neurosci. 27, 421–434. doi: 10.1515/revneuro-2015-0053, PMID: 26756089

[ref9] Buhl D. L. Harris K. D. Hormuzdi S. G. Monyer H. Buzsáki G. (2003). Selective impairment of hippocampal gamma oscillations in connexin-36 knock-out mouse in vivo. J. Neurosci. 23, 1013–1018. doi: 10.1523/JNEUROSCI.23-03-01013.2003, PMID: 12574431 PMC6741916

[ref10] Chenot Q. Hamery C. Truninger M. Langer N. De Boissezon X. Scannella S. (2024). Investigating the relationship between resting-state EEG microstates and executive functions: a null finding. Cortex 178, 1–17. doi: 10.1016/j.cortex.2024.05.019, PMID: 38954985

[ref11] Coulon P. Landisman C. E. (2017). The potential role of gap junctional plasticity in the regulation of state. Neuron 93, 1275–1295. doi: 10.1016/j.neuron.2017.02.041, PMID: 28334604

[ref12] Csorba B. A. Krause M. R. Zanos T. P. Pack C. C. (2022). Long-range cortical synchronization supports abrupt visual learning. Curr. Biol. 32, 2467–2479.e4. doi: 10.1016/j.cub.2022.04.029, PMID: 35523181

[ref13] Cunningham M. O. Whittington M. A. Bibbig A. Roopun A. LeBeau F. E. N. Vogt A.. (2004). A role for fast rhythmic bursting neurons in cortical gamma oscillations in vitro. Proc. Natl. Acad. Sci. USA 101, 7152–7157. doi: 10.1073/pnas.0402060101, PMID: 15103017 PMC406481

[ref14] Cutting N. Apperly I. A. Chappell J. Beck S. R. (2014). The puzzling difficulty of tool innovation: why can’t children piece their knowledge together? J. Exp. Child Psychol. 125, 110–117. doi: 10.1016/j.jecp.2013.11.010, PMID: 24530037

[ref15] Defeyter M. A. German T. P. (2003). Acquiring an understanding of design: evidence from children's insight problem solving. Cognition 89, 133–155. doi: 10.1016/S0010-0277(03)00098-2, PMID: 12915298

[ref16] Dere E. (2013). Gap junctions in the brain: physiological and pathological roles. San Diego, CA: Academic Press.

[ref17] Dere E. Kart-Teke E. Huston J. P. De Souza Silva M. A. (2006). The case for episodic memory in animals. Neurosci. Biobehav. Rev. 30, 1206–1224. doi: 10.1016/j.neubiorev.2006.09.005 17079013

[ref18] Dere E. Pause B. Pietrowsky R. (2010). Emotion and episodic memory in neuropsychiatric disorders. Behav. Brain Res. 215, 162–171. doi: 10.1016/j.bbr.2010.03.017 20227444

[ref19] Dere E. Zlomuzica A. (2012). The role of gap junctions in the brain in health and disease. Neurosci. Biobehav. Rev. 36, 206–217. doi: 10.1016/j.neubiorev.2011.05.015, PMID: 21664373

[ref20] Dere E. Zlomuzica A. (2023). Editorial: special issue on the neuroscience of consciousness. Behav. Brain Res. 437:114166. doi: 10.1016/j.bbr.2022.114166 36270463

[ref21] Dere D. Zlomuzica A. Dere E. (2019). Fellow travellers in cognitive evolution: co-evolution of working memory and mental time travel? Neurosci. Biobehav. Rev. 105, 94–105. doi: 10.1016/j.neubiorev.2019.07.016, PMID: 31381932

[ref22] Dere D. Zlomuzica A. Dere E. (2021). Channels to consciousness: a possible role of gap junctions in consciousness. Rev. Neurosci. 32, 101–129. doi: 10.1515/revneuro-2020-0012, PMID: 32853172

[ref23] Dere E. Zlomuzica A. Huston J. P. De Souza Silva M. A. (2008). “Chapter 2.2: animal episodic memory” in Handbook of episodic memory. eds. Dere E. Easton A. Nadel L. Huston J. P., vol. 18 (Amsterdam: Elsevier Science), 155–184.

[ref24] Draguhn A. Traub R. D. Schmitz D. Jefferys J. G. (1998). Electrical coupling underlies high-frequency oscillations in the hippocampus in vitro. Nature 394, 189–192. doi: 10.1038/28184, PMID: 9671303

[ref25] Durstewitz D. Vittoz N. M. Floresco S. B. Seamans J. K. (2010). Abrupt transitions between prefrontal neural ensemble states accompany behavioral transitions during rule learning. Neuron 66, 438–448. doi: 10.1016/j.neuron.2010.03.029, PMID: 20471356

[ref26] Ebel S. Völter C. Call J. (2020). Prior experience mediates the usage of food items as tools in great apes (pan paniscus, pan troglodytes, Gorilla gorilla, and *Pongo abelii*). J. Comp. Psychol. 135, 64–73. doi: 10.1037/com0000236, PMID: 32463250

[ref27] Edlow B. L. Claassen J. Schiff N. D. Greer D. M. (2021). Recovery from disorders of consciousness: mechanisms, prognosis and emerging therapies. Nat. Rev. Neurol. 17, 135–156. doi: 10.1038/s41582-020-00428-x, PMID: 33318675 PMC7734616

[ref28] Frisch C. De Souza-Silva M. A. Söhl G. Güldenagel M. Willecke K. Huston J. P.. (2005). Stimulus complexity dependent memory impairment and changes in motor performance after deletion of the neuronal gap junction protein connexin36 in mice. Behav. Brain Res. 157, 177–185. doi: 10.1016/j.bbr.2004.06.023, PMID: 15617784

[ref29] Gallistel C. R. Fairhurst S. Balsam P. (2004). The learning curve: implications of a quantitative analysis. Proc. Natl. Acad. Sci. USA 101, 13124–13131. doi: 10.1073/pnas.0404965101, PMID: 15331782 PMC516535

[ref30] Hebb D. O. (1949). The organization of behaviour: A neuropsychological theory. New York: Wiley.

[ref31] Helson H. (1927). Insight in the white rat. J. Exp. Psychol. 10, 378–396. doi: 10.1037/h0070577

[ref32] Hormuzdi S. G. Pais I. LeBeau F. E. Towers S. K. Rozov A. Buhl E. H.. (2001). Impaired electrical signaling disrupts gamma frequency oscillations in connexin 36-deficient mice. Neuron 31, 487–495. doi: 10.1016/S0896-6273(01)00387-7, PMID: 11516404

[ref33] Jung-Beeman M. Bowden E. M. Haberman J. Frymiare J. L. Arambel-Liu S. Greenblatt R.. (2004). Neural activity when people solve verbal problems with insight. PLoS Biol. 2:E97. doi: 10.1371/journal.pbio.0020097, PMID: 15094802 PMC387268

[ref34] Kinugawa K. Schumm S. Pollina M. Depre M. Jungbluth C. Doulazmi M.. (2013). Aging-related episodic memory decline: are emotions the key? Front. Behav. Neurosci. 7:2. doi: 10.3389/fnbeh.2013.00002 23378831 PMC3561617

[ref35] Knoblich G. Ohlsson S. Raney G. E. (2001). An eye movement study of insight problem solving. Mem. Cogn. 29, 1000–1009. doi: 10.3758/BF03195762 11820744

[ref36] Koch C. Massimini M. Boly M. Tononi G. (2016). Neural correlates of consciousness: progress and problems. Nat. Rev. Neurosci. 17, 307–321. doi: 10.1038/nrn.2016.22, PMID: 27094080

[ref37] Kocsis B. Martínez-Bellver S. Fiáth R. Domonkos A. Sviatkó K. Schlingloff D.. (2022). Huygens synchronization of medial septal pacemaker neurons generates hippocampal theta oscillation. Cell Rep. 40:111149. doi: 10.1016/j.celrep.2022.111149 35926456

[ref38] Köhler W. (1917). Intelligenzprüfungen an Anthropoiden. Berlin: Royal Prussian Society of Sciences.

[ref39] Köhler W. (1925). The mentality of apes. Oxford, England: Harcourt, Brace.

[ref40] Kounios J. Beeman M. (2014). The cognitive neuroscience of insight. Annu. Rev. Psychol. 65, 71–93. doi: 10.1146/annurev-psych-010213-115154 24405359

[ref41] Landisman C. E. Long M. A. Beierlein M. Deans M. R. Paul D. L. Connors B. W. (2002). Electrical synapses in the thalamic reticular nucleus. J. Neurosci. 22, 1002–1009. doi: 10.1523/JNEUROSCI.22-03-01002.2002, PMID: 11826128 PMC6758490

[ref42] Laumer I. B. Call J. Bugnyar T. Auersperg A. M. I. (2018). Spontaneous innovation of hook-bending and unbending in orangutans (*Pongo abelii*). Sci. Rep. 8:16518. doi: 10.1038/s41598-018-34607-0 30410111 PMC6224503

[ref43] Laureys S. Celesia G. G. Cohadon F. Lavrijsen J. León-Carrión J. Sannita W. G.. (2010). European task force on disorders of consciousness. Unresponsive wakefulness syndrome: a new name for the vegetative state or apallic syndrome. BMC Med. 8:68. doi: 10.1186/1741-7015-8-68, PMID: 21040571 PMC2987895

[ref44] Le Bon-Jego M. Yuste R. (2007). Persistently active, pacemaker-like neurons in neocortex. Front. Neurosci. 1, 123–129. doi: 10.3389/neuro.01.1.1.009.2007, PMID: 18982123 PMC2518052

[ref45] Li H. Zhang X. Sun X. Dong L. Lu H. Yue S.. (2023). Functional networks in prolonged disorders of consciousness. Front. Neurosci. 17:1113695. doi: 10.3389/fnins.2023.1113695, PMID: 36875660 PMC9981972

[ref46] Linsambarth S. Carvajal F. J. Moraga-Amaro R. Mendez L. Tamburini G. Jimenez I.. (2022). Astroglial gliotransmitters released via Cx43 hemichannels regulate NMDAR-dependent transmission and short-term fear memory in the basolateral amygdala. FASEB J. 36:e22134. doi: 10.1096/fj.202100798RR, PMID: 35061296

[ref47] MacGregor J. N. Ormerod T. C. Chronicle E. P. (2001). Information processing and insight: a process model of performance on the nine-dot and related problems. J. Exp. Psychol. Learn. Mem. Cogn. 27, 176–201. doi: 10.1037/0278-7393.27.1.176, PMID: 11204097

[ref48] Maciunas K. Snipas M. Paulauskas N. Bukauskas F. F. (2016). Reverberation of excitation in neuronal networks interconnected through voltage-gated gap junction channels. J. Gen. Physiol. 147, 273–288. doi: 10.1085/jgp.201511488, PMID: 26880752 PMC4772373

[ref49] Mäkinen M. E. Ylä-Outinen L. Narkilahti S. (2018). GABA and gap junctions in the development of synchronized activity in human pluripotent stem cell-derived neural networks. Front. Cell. Neurosci. 12:56. doi: 10.3389/fncel.2018.00056, PMID: 29559893 PMC5845705

[ref50] Martin S. (2023). Why using “consciousness” in psychotherapy? Insight, metacognition and self-consciousness. New Ideas Psychol. 70:101015. doi: 10.1016/j.newideapsych.2023.101015

[ref51] McCaffrey T. (2012). Innovation relies on the obscure: a key to overcoming the classic problem of functional fixedness. Psychol. Sci. 23, 215–218. doi: 10.1177/0956797611429580, PMID: 22318998

[ref52] Meador K. J. Ray P. G. Echauz J. R. Loring D. W. Vachtsevanos G. J. (2002). Gamma coherence and conscious perception. Neurology 59, 847–854. doi: 10.1212/WNL.59.6.847 12297565

[ref53] Meier C. Petrasch-Parwez E. Habbes H. W. Teubner B. Güldenagel M. Degen J.. (2002). Immunohistochemical detection of the neuronal connexin36 in the mouse central nervous system in comparison to connexin36-deficient tissues. Histochem. Cell Biol. 117, 461–471. doi: 10.1007/s00418-002-0417-z 12107497

[ref54] Nagy J. I. Dudek F. E. Rash J. E. (2004). Update on connexins and gap junctions in neurons and glia in the mammalian nervous system. Brain Res. Brain Res. Rev. 47, 191–215. doi: 10.1016/j.brainresrev.2004.05.005, PMID: 15572172

[ref55] Northoff G. (2017). "paradox of slow frequencies"—are slow frequencies in upper cortical layers a neural predisposition of the level/state of consciousness (NPC)? Conscious. Cogn. 54, 20–35. doi: 10.1016/j.concog.2017.03.006, PMID: 28392004

[ref56] Panagiotaropoulos T. I. Deco G. Kapoor V. Logothetis N. K. (2012). Neuronal discharges and gamma oscillations explicitly reflect visual consciousness in the lateral prefrontal cortex. Neuron 74, 924–935. doi: 10.1016/j.neuron.2012.04.013, PMID: 22681695

[ref57] Panda R. Thibaut A. Lopez-Gonzalez A. Escrichs A. Bahri M. A. Hillebrand A.. (2022). Disruption in structural-functional network repertoire and time-resolved subcortical fronto-temporoparietal connectivity in disorders of consciousness. eLife 11:e77462. doi: 10.7554/eLife.77462, PMID: 35916363 PMC9385205

[ref58] Pause B. M. Zlomuzica A. Kinugawa K. Mariani J. Pietrowsky R. Dere E. (2013). Perspectives on episodic-like and episodic memory. Front. Behav. Neurosci. 7:33. doi: 10.3389/fnbeh.2013.00033 23616754 PMC3629296

[ref59] Qiu J. Li H. Yang D. Luo Y. Li Y. Wu Z.. (2008). The neural basis of insight problem solving: an event-related potential study. Brain Cogn. 68, 100–106. doi: 10.1016/j.bandc.2008.03.004, PMID: 18433966

[ref60] Rash J. E. Yasumura T. Dudek F. E. Nagy J. I. (2001). Cell-specific expression of connexins and evidence of restricted gap junctional coupling between glial cells and between neurons. J. Neurosci. 21, 1983–2000. doi: 10.1523/JNEUROSCI.21-06-01983.2001, PMID: 11245683 PMC1804287

[ref61] Reddy G. (2022). A reinforcement-based mechanism for discontinuous learning. Proc. Natl. Acad. Sci. USA 119:e2215352119. doi: 10.1073/pnas.2215352119, PMID: 36442113 PMC9894243

[ref62] Rosen A. Reiner M. (2017). Right frontal gamma and beta band enhancement while solving a spatial puzzle with insight. Int. J. Psychophysiol. 122, 50–55. doi: 10.1016/j.ijpsycho.2016.09.008, PMID: 27671505

[ref63] Rosenberg M. Zhang T. Perona P. Meister M. (2021). Mice in a labyrinth show rapid learning, sudden insight, and efficient exploration. eLife 10:e66175. doi: 10.7554/eLife.66175, PMID: 34196271 PMC8294850

[ref64] Sandkühler S. Bhattacharya J. (2008). Deconstructing insight: EEG correlates of insightful problem solving. PLoS One 3:e1459. doi: 10.1371/journal.pone.0001459, PMID: 18213368 PMC2180197

[ref65] Schmitz D. Schuchmann S. Fisahn A. Draguhn A. Buhl E. H. Petrasch-Parwez E.. (2001). Axo-axonal coupling. A novel mechanism for ultrafast neuronal communication. Neuron 31, 831–840. doi: 10.1016/S0896-6273(01)00410-X, PMID: 11567620

[ref66] Schultz W. Dayan P. Montague P. R. (1997). A neural substrate of prediction and reward. Science 275, 1593–1599. doi: 10.1126/science.275.5306.1593 9054347

[ref67] Shen W. Tong Y. Yuan Y. Zhan H. Liu C. Luo J.. (2017). Feeling the insight: uncovering somatic markers of the “aha” experience. Appl. Psychophysiol. Biofeedback 43, 13–21. doi: 10.1007/s10484-017-9381-1, PMID: 29075938

[ref68] Singer W. (1998). Consciousness and the structure of neuronal representations. Philos. Trans. R. Soc. Lond. Ser. B Biol. Sci. 353, 1829–1840. doi: 10.1098/rstb.1998.0335, PMID: 9854255 PMC1692413

[ref69] Stefanelli R. (2023). Theories of consciousness and psychiatric disorders—a comparative analysis. Neurosci. Biobehav. Rev. 152:105204. doi: 10.1016/j.neubiorev.2023.105204, PMID: 37127069

[ref70] Tao X. D. Liu Z. R. Zhang Y. Q. Zhang X. H. (2021). Connexin43 hemichannels contribute to working memory and excitatory synaptic transmission of pyramidal neurons in the prefrontal cortex of rats. Life Sci. 286:120049. doi: 10.1016/j.lfs.2021.120049, PMID: 34662549

[ref71] Thorndike E. L. (1911). Animal Intelligence: Experimental Studies. Lewiston, NY, United States: Macmillan Press.

[ref72] Thorpe W. H. (1956). Learning and instinct in animals. Cambridge, MA: Harvard University Press.

[ref73] Tian Y. Margulies D. S. Breakspear M. Zalesky A. (2020). Topographic organization of the human subcortex unveiled with functional connectivity gradients. Nat. Neurosci. 23, 1421–1432. doi: 10.1038/s41593-020-00711-6, PMID: 32989295

[ref74] Tik M. Sladky R. Luft C. D. B. Willinger D. Hoffmann A. Banissy M. J.. (2018). Ultra-high-field fMRI insights on insight: neural correlates of the Aha!-moment. Hum. Brain Mapp. 39, 3241–3252. doi: 10.1002/hbm.24073, PMID: 29665228 PMC6055807

[ref75] Tolman E. C. (1948). Cognitive maps in rats and men. Psychol. Rev. 55, 189–208. doi: 10.1037/h0061626, PMID: 18870876

[ref76] Tononi G. Boly M. Massimini M. Koch C. (2016). Integrated information theory: from consciousness to its physical substrate. Nat. Rev. Neurosci. 17, 450–461. doi: 10.1038/nrn.2016.44, PMID: 27225071

[ref77] Traub R. D. Whittington M. A. (2010). Cortical oscillations in health and disease. New York: Oxford Univ. Press.

[ref78] Traub R. D. Whittington M. A. (2022). Processing of cell assemblies in the lateral entorhinal cortex. Rev. Neurosci. 33, 829–847. doi: 10.1515/revneuro-2022-0011, PMID: 35447022

[ref79] Traub R. D. Whittington M. A. Gutiérrez R. Draguhn A. (2018). Electrical coupling between hippocampal neurons: contrasting roles of principal cell gap junctions and interneuron gap junctions. Cell Tissue Res. 373, 671–691. doi: 10.1007/s00441-018-2881-3, PMID: 30112572

[ref80] Traub R. D. Whittington M. A. Maier N. Schmitz D. Nagy J. I. (2020). Could electrical coupling contribute to the formation of cell assemblies? Rev. Neurosci. 31, 121–141. doi: 10.1515/revneuro-2019-0059, PMID: 31536035

[ref81] Tseng P. Chang Y. T. Chang C. F. Liang W. K. Juan C. H. (2016). The critical role of phase difference in gamma oscillation within the temporoparietal network for binding visual working memory. Sci. Rep. 6:32138. doi: 10.1038/srep32138 27573864 PMC5004173

[ref82] Valencia A. L. Froese T. (2020). What binds us? Inter-brain neural synchronization and its implications for theories of human consciousness. Neurosci. Conscious 2020:niaa010. doi: 10.1093/nc/niaa010, PMID: 32547787 PMC7288734

[ref83] Van Steenburgh J. J. Fleck J. I. Beeman M. Kounios J. (2012). Insight. The Oxford handbook of thinking and reasoning. United Kingdom: Oxford University Press.

[ref84] Walrave L. Vinken M. Albertini G. De Bundel D. Leybaert L. Smolders I. J. (2016). Inhibition of Connexin43 Hemichannels impairs spatial short-term memory without affecting spatial working memory. Front. Cell. Neurosci. 10:288. doi: 10.3389/fncel.2016.00288 28066184 PMC5168429

[ref85] Weir A. A. S. (2002). Shaping of hooks in new Caledonian crows. Science 297:981. doi: 10.1126/science.1073433, PMID: 12169726

[ref86] Weisberg R. W. (2015). Toward an integrated theory of insight in problem solving. Think. Reason. 21, 5–39. doi: 10.1080/13546783.2014.886625

[ref87] Wittner L. Miles R. (2007). Factors defining a pacemaker region for synchrony in the hippocampus. J. Physiol. 584, 867–883. doi: 10.1113/jphysiol.2007.138131, PMID: 17823211 PMC2276992

[ref88] Yang X. D. Korn H. Faber D. S. (1990). Long-term potentiation of electrotonic coupling at mixed synapses. Nature 348, 542–545. doi: 10.1038/348542a0 2174130

[ref89] Zlomuzica A. Dere E. (2022). Towards an animal model of consciousness based on the platform theory. Behav. Brain Res. 419:113695. doi: 10.1016/j.bbr.2021.113695, PMID: 34856300

[ref90] Zlomuzica A. Plank L. Dere E. (2022). A new path to mental disorders: through gap junction channels and hemichannels. Neurosci. Biobehav. Rev. 142:104877. doi: 10.1016/j.neubiorev.2022.104877, PMID: 36116574

[ref91] Zlomuzica A. Plank L. Kodzaga I. Dere E. (2023). A fatal alliance: glial connexins, myelin pathology and mental disorders. J. Psychiatr. Res. 159, 97–115. doi: 10.1016/j.jpsychires.2023.01.008, PMID: 36701970

[ref92] Zlomuzica A. Reichinnek S. Maxeiner S. Both M. May E. Wörsdörfer P.. (2010). Deletion of connexin45 in mouse neurons disrupts one-trial object recognition and alters kainate-induced g-oscillations in the hippocampus. Physiol. Behav. 101, 245–253. doi: 10.1016/j.physbeh.2010.05.007, PMID: 20471991

[ref93] Zlomuzica A. Viggiano D. Degen J. Binder S. Ruocco L. A. Sadile A. G.. (2012). Behavioral alterations and changes in Ca/calmodulin kinase II levels in the striatum of connexin36 deficient mice. Behav. Brain Res. 226, 293–300. doi: 10.1016/j.bbr.2011.08.028, PMID: 21889545

